# Which of 51 Plate Designs Can Most Stably Fixate the Fragments in a Fracture of the Mandibular Condyle Base?

**DOI:** 10.3390/jcm12134508

**Published:** 2023-07-05

**Authors:** Marcin Kozakiewicz, Jakub Okulski, Michał Krasowski, Bartłomiej Konieczny, Rafał Zieliński

**Affiliations:** 1 Department of Maxillofacial Surgery, Medical University of Lodz, 113 Żeromskiego Str., 90-549 Lodz, Poland; jakub.okulski@umed.lodz.pl (J.O.); bkost@op.pl (R.Z.); 2 Material Science Laboratory, Medical University of Lodz, 251 Pomorska Str., 92-213 Lodz, Poland; michal.krasowski@umed.lodz.pl (M.K.); bartlomiej.konieczny@umed.lodz.pl (B.K.)

**Keywords:** fixing material, osteosynthesis, surgical treatment, fracture fixation, open reduction internal fixation, mandible condyle, plate, condylar, basal

## Abstract

In the surgical treatment of the most common fracture of the mandible, which is a fracture of the condylar base, a great choice of different plate shapes is observed. The aim of this study was to determine which shape gives the greatest fixation stiffness. To ensure homogeneity in comparison, tests were performed on polyurethane models divided at the level of the condylar base fracture and each were fixed with 51 plates. The plates were cut from a 1 mm thick grade 23 titanium sheet. The models were then loaded and the force required for 1 mm of fracture displacement was recorded. It was noted that in addition to osteosynthesis from two simple plates, there were also two dedicated single plates with similar rigidity. Among the large number of described designs of plates, there is considerable variation in terms of the stability of the fixation performed with them. The proposed Mechanical Excellence Factor allows a pre-evaluation of the expected rigidity of fixation with a given plate shape without the need for a loading experiment. The authors expect this to be helpful for surgeons in the application of relevant plates, as well for inventors of new plates for the osteosynthesis of basal fractures in mandibular condyle.

## 1. Introduction

Mandibular fracture is one of the most common facial bone injuries [1] and is a trauma in which oral and maxillofacial surgeons are important for the first aid, final treatment and management of late complications. Undoubtedly, such injuries involve fractures of the condylar process of the mandible, including its base fracture, which are the most common fractures of the mandibular condyle [2,3]. Therefore, these fractures of the base of the condylar process have been treated for decades [4]. This was due to the relatively easy surgical access (previously it was submandibular) [4,5,6], but the fixing material was quite imperfect. Initially, bone wiring was used [7,8,9], and in the 1950s experiments began with plates [10,11], but it was not until the 1980s that plate osteosynthesis became widespread [12]. In the meantime, in the 1960s, extraoral pins were used for fixation [13]. The introduction of direct-compression osteosynthesis was initiated in the 1970s [14]. The straight plates were made of various metal alloys [15,16,17,18]. Compression plates made of stainless steel [15] have given way to smaller miniplates made of titanium [19], and in the 1990s those plates became popular [20,21,22,23,24,25]. Then came the era of technical facilitation in operating on this difficult anatomical region—dedicated plates were created. The first to be created were squares, rectangles and deltas. Nowadays, they are treated in a number of surgical ways using a wide collection of dedicated titanium plates [26,27,28,29,30,31,32]. This plethora of choices raises the question of whether, therefore, each plate is equally suitable for treating fractures of the base of the condylar process. And in addition, there have recently been descriptions of new dedicated plates for fractures of the condylar process of the mandible [33,34,35,36,37,38]. Therefore, the state of the art of open reduction and internal fixation with titanium plates has been achieved. There are now dozens of these types of plates.

The aim of the study was to verify which design among 51 known plates most stably fixes fracture of the base of the condylar process of the mandible.

## 2. Materials and Methods

Most of the designs examined are for plates recommended by the developers and manufacturers for fixation of mandibular condylar process fractures. Some do not have this qualification but can easily be used to perform osteosynthesis of mandibular condyle fractures. They were included in the study in the hope that these shape designs would prove rigid enough for osteosynthesis of this fracture and indicate good new mechanical solutions for traumatology patients. Thus, 51 plate designs were selected (Table 1). In the first stage, 7 copies of each shape were made from titanium sheet, in grade 23 alloy certified for medical implants. Thickness of the titanium material was 1 mm.

**Figure 1:**
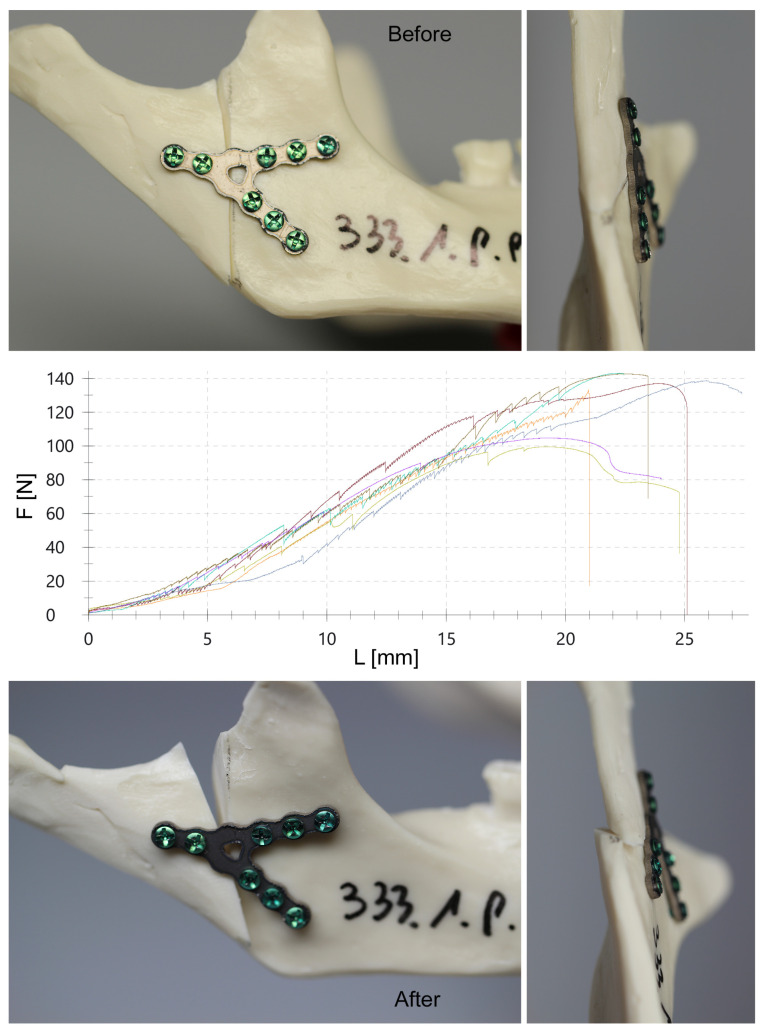
Polyurethane model of mandibular condylar process base fracture fixated with “Y geometry” plate [34]. Average result for that plate is 5.83 ± 0.52 N/mm. This particular experiment corresponds to the brown line on the graph (Fmax = 143 N and the length of the displacement distance is 23.4 mm). Each line in the plot represents each of the 7 plates included in the study in a given group.

Solid polyurethane foam mandibles were utilized in this study (Figure 1). The high variability in the density and the elastic modulus of bone affects biomechanical testing results [39]. Synthetic foam materials have been shown to produce less intra- and inter- specimen variability than cadaver bone [40]. A foam block has consistent material properties, similar to human cancellous bone. Solid polyurethane foam is widely used as an ideal medium to mimic human cancellous bone and has been confirmed by the American Society for Testing and Materials [41,42] as a standard material for testing orthopedic devices and instruments. In this study, polyurethane foam (Sawbones, Vashon, WA, USA: density 0.16 g/cc, compression modulus 58 MPa) was used as a substitute for bone [43,44,45,46].

The polyurethane models were then cut at the level of the condylar process base fracture [3,47]. Each such fracture was fixated with previously prepared plates. Predrilling was performed with a 1.5 mm drill. Each hole in the plates was filled by screws. All plates were fixed by self-tapping screws of 6 mm length and 2 mm external diameter. Plate models were grouped with seven plates for each design (i.e., each design was tested seven times). Thus, after fixation, 357 experimental condylar osteosynthesis were obtained.

The condyles were set at a 15° inferior tilt in the sagittal plane and at a 10° lateral tilt in the coronal plane to simulate actual masticatory force loading on the temporomandibular joint. This model results in the condyle exerting a force upwards and somewhat forwards and medially [48].

For testing purposes, Zwick Roell Z020 universal strength machine (Zwick-Roell, Ulm, Germany) with an individually made clamping system was used. Clamping system comprised flat 1 mm thick stainless steel based on 70 cm × 60 cm angulated aluminum block with milled 4 × M6 threaded holes for screwing the flat base plate. On the plate for stabilization of mandible, stainless steel try square was used. Pre-load force was 1 N and test speed was 1 mm/min. The action point of the compressive forces was located at the condyle. The load vs. displacement relationship, load for permanent deformation and maximum load at fracture were recorded using the lnstron chart recorder (testXpert II V3.31, Zwick Roell, Ulm, Germany). Permanent deformation was defined as the initial point that the load–displacement relationship was no longer linear. Maximum load was defined as the greatest load recorded just before any sudden decrease in load level.

The following features derived from the designs of the plates were noted: Number of Screws in Ramus, Number of Screws in Condyle, Total Fixing Screw Number, Height (mm), Width (mm), Plate Surface Area (mm^2^), % Round Holes (i.e., the percentage of circular holes in the plate among all holes designed in the plate), Number of Oval Holes in Plate (i.e., the number of oval holes designed in the plate), Oval Holes Share (the ratio of the number of oval holes for screws to the number of round holes designed in the plate).

In addition to the already known Plate Design Factor (PDF) [29,49,50], it was found to be statistically feasible to develop a new indicator describing, by a single number, numerous features of the plate design, incorporating more characteristic aspects of the design than the PDF: the Mechanical Excellence Factor (MEF). MEF was next calculated for each plate (Table 1).

The statistics analysis was performed in Statgraphics Centurion 18 (Statgraphics Technologies Inc. The Plains City, Warrenton, VA, USA). The ANOVA or Kruskal–Wallis test was applied for between-design comparisons. Independent χ^2^ tests were used to test the categorical variables. The relationship between the two quantitative variables was assessed by linear regression analysis. The best plate design was indicated based on objective description. A *p*-value less than 0.05 was considered statistically significant.

## 3. Results

**Figure 2:**
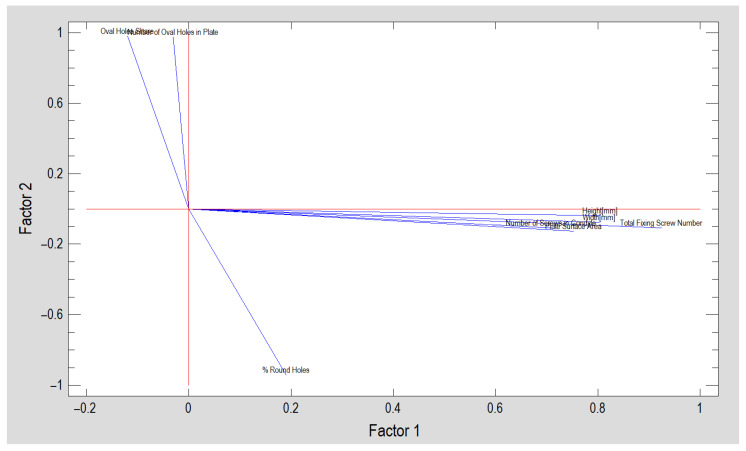
Factor analysis—factor loadings. By evaluating factor loadings, one can understand what the factors mean and name them. Therefore, Factor 1 can be called an improvement component and Factor 2 a deteriorating component of plate mechanical excellence based on 8 plate design features. Name of used variable presented in the plot: Total Fixing Screw Number, Number of Screws in Condyle, Height[mm], Width[mm], Plate Surface Area, % Round Holes, Number of Oval Holes in Plate and Oval Holes Share.

The purpose of the factor analysis is to obtain a small number of factors which account for most of the variability in the eight variables. In this case, two factors have been extracted, since two factors had eigenvalues greater than or equal to 1.0 (Figure 2). Together, they account for 74.6% of the variability in the original data. Input data variables were Total Fixing Screw Number, Number of Screws in Condyle, Height (mm), Width (mm), Plate Surface Area (mm^2^), Percentage of Round Holes in Plate, Number of Oval Holes in Plate and Oval Holes Share. A factorability test (Kaiser–Meyer–Olkin Measure of Sampling Adequacy, KMO) was performed to provide indications of whether or not it is likely to be worthwhile attempting to extract factors from a set of variables. The KMO statistic provides an indication of how much common variance is present. For factorization to be worthwhile, KMO should normally be at least 0.6. and, here, KMO = 0.628, thus factorization is likely to provide interesting information about any underlying factors.

Next, factor loading rotation was performed in order to simplify the explanation of the factors. The rotated factors have the following equations (the coefficients in the equations correspond to the places on the graph plane in Figure 2 indicated by the factor loadings):

*Factor* 1 = *Improvement Component* = 0.924362∙*Total Fixing Screw Number* + 0.708092∙*Number of Screws in Condyle* + 0.804335∙*Height*[mm] + 0.802964∙Width[mm] + 0.752599∙*Plate Surface Area* + 0.189877% *Round Holes* − 0.0300946∙*Number of Oval Holes in Plate* − 0.11967∙*Oval Holes Share* (1)

*Factor* 2 = *Deteriorating Component* = −0.108505∙*Total Fixing Screw Number* − 0.109∙*Number of Screws in Condyle* − 0.0375889∙*Height*[mm] − 0.0761193∙*Width*[mm] − 0.127803∙*Plate Surface Area* − 0.940781% *Round Holes* + 0.97506∙*Number of Oval Holes in Plate* + 0.980953∙*Oval Holes Share* (2)

where the values of the variables in the equation are standardized by subtracting their means and dividing by their standard deviations.

Because of the opposite meaning of the loadings in Factor 1 versus Factor 2, a second-order factor analysis was performed. In this way, a single feature (MEF) combining information on eight features of the design of the mandibular condylar process fracture fixation plate was obtained (it accounts for 86.5% of the variability in the original data from Factor 1 and Factor 2). The distribution of the factor calculated in this way in the experimental data shows a slight skewness (standardized skewness is 3.854), so to normalize the distribution it was transformed by rooting—a normal distribution was obtained (standardized skewness = 0.089 and standardized kurtosis = −1.564). This is how MEF is constructed in its own way:

(3) $$Mechanical\;Excellence\;Factor=\sqrt[{2}]{0.930016\cdot Improvement\;Component-0.930016\cdot Deteriorating\;Component}$$

**Figure 3:**
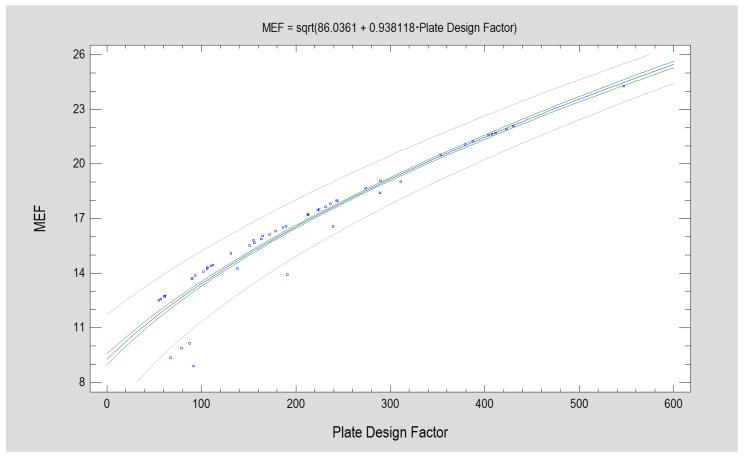
Mechanical Excellence Factor (MEF) is related to Plate Design Factor (*p* < 0.001). The two characteristics are relatively strongly related (correlation coefficient, CC = 0.95) and the presented mathematical description model describes a significant majority of observations (R^2^ = 95.1). The regression equation plot is shown by the blue line. The green lines constrain the confidence limits (for a 95% confidence level). And gray lines constrain the prediction limits. A decrease in the relationship of both factors is observed for designs with lower PDF and MEF values.

MEF has a direct proportional relationship with the Plate Design Factor (Figure 3), with which it describes the mechanical strength of the structure. The authors are interested in introducing a measure called MEF because it incorporates more features of plate design than the known Plate Design Factor.

In this way, a numerical description of the designs of each plate was obtained. Table 1 below shows the obtained fixation stability results of each of the 51 titanium plate models. The reported average force displacing the condylar process fixation by 1 mm was 5.75 ± 3.77 N and did not have a normal distribution. The median was 5.14 N. Compared to the load of the intact mandibular model (28.33 ± 3.16 N), this value is significantly lower (Mann–Whitney (Wilcoxon) test W = 2499; *p* < 0.001).

**Figure 4:**
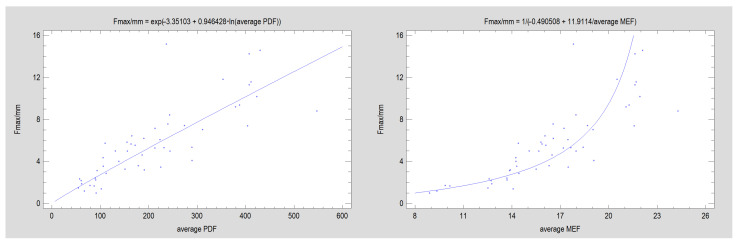
Relationship of experimental results to numerical description of plate design (*p* < 0.001). The graphs show the average, resulting from seven repetitions of load of the same design plate; (**left**) Plate Design Factor, PDF, and (**right**) Mechanical Excellence Factor, MEF, calculated for each of the 51 experimental groups. Each group represents 1 result in the graph.

Note (Figure 4) that the average experimental value of Fmax/dL for plate design corresponds to the MEF calculated from the design (CC = 0.90; R^2^ = 80%; *p* < 0.001). PDF is weaker in relation to Fmax/dL among the tested plated designs (CC = 0.86; R^2^ = 74%; *p* < 0.001). The number and share of oval holes in the structure of the plate are construction features that occur more often in relation to the lower forces that displaced the fixed fragments.

**Figure 5:**
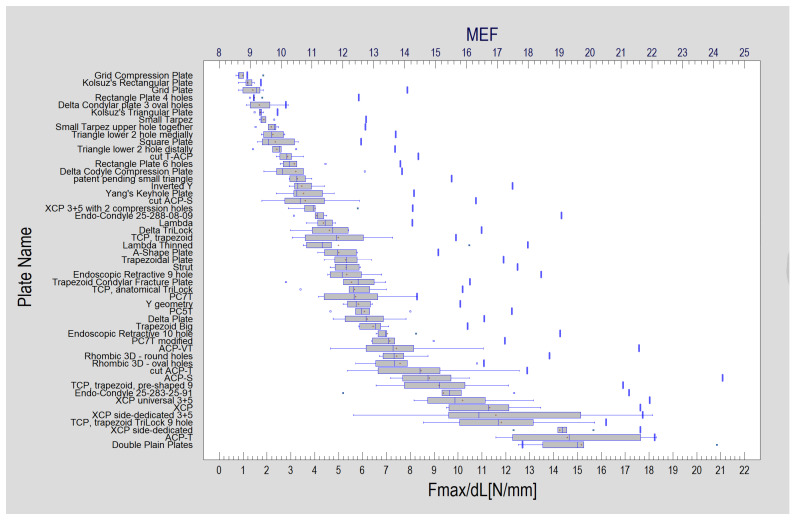
Experimental results for each plate design. Forces needed for one-millimeter displacement of the fixed fragments: mean (red cross), median (thin vertical line inside the grey box). And on the top axis described in dark blue is the Mechanical Excellence Factor (MEF), reflected in the graph as thick vertical lines. The general trend of increasing plate rigidity with increasing MEF can be seen.

The results of the amount of force displacing the fixation by 1 mm between all tested plate designs showed significant differences (Kruskal–Wallis test, test statistic = 327; *p* < 0.001). The highest result was recorded for two-plate osteosynthesis with straight plates (15.17 ± 2.69 N). However, it was detected that the fixations by the ACP-T (plate 23) or XCP side-dedicated (plate 10) were not statistically weaker than double plain plate osteosynthesis (Figure 5 and Table 2). 

**Figure 6:**
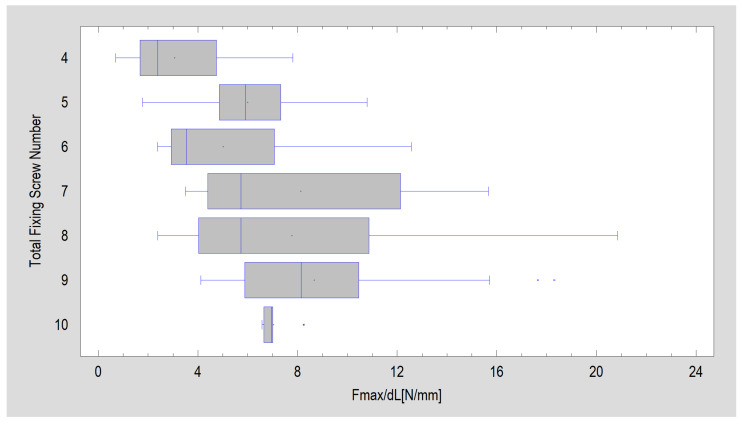
Forces needed for one-millimeter displacement of the fixed fragments in designs grouped by total number of fixing screws: mean (red cross), median (thin vertical line inside the box). The data apply only to dedicated plates—there are no data for two-plate osteosynthesis.

In dedicated plates (excluding double plain plating), increasing the number of screws fixing the plate to the fracture fragments of the mandibular condylar process increases the rigidity of fixation. The best results were obtained with plates having 7–10 fixation holes (Kruskal–Wallis test, test statistics = 159; *p* < 0.001). The highest stability of fixation (i.e., highest displacement forces of 1 mm) was achieved for plates fixed with nine screws (8.66 ± 3.34 N/mm). All the collected results for the number of screws are shown in Figure 6.

**Figure 7:**
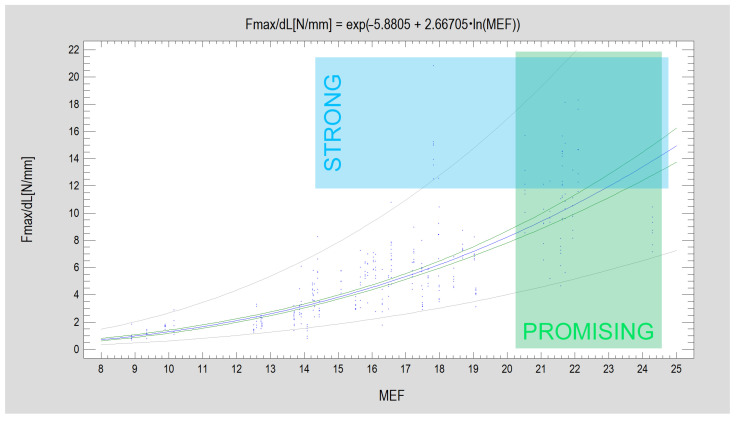
Relationship of cumulative evaluation of plate construction (Mechanical Excellence Factor, MEF) to resistance to displacement force of fixated fragments of mandibular condylar process base fracture (*p* < 0.001). Above data present all experimental results collected (357 experiments). The two characteristics are relatively strongly related (CC = 0.86) and the presented mathematical description model describes a significant majority of observations (R^2^ = 73%). The regression equation plot is shown by the blue line. The points represent the experimental results obtained. The exact results of the load tests are shown in Table 1. The green lines constrain the confidence limits (for a 95% confidence level). And gray lines constrain the prediction limits. A group of designs with promising construction features (green rectangle signed PROMISING) and a group of designs that enable fixation with high stability (blue rectangle signed STRONG) are noticeable. The contents of the two sets partially overlap.

Plate designs with an MEF higher than 20 are classified as promising plate designs. And designs that passed tests achieving a force displacing segments of 1 mm above 12 N were classified as strong plate designs (Figure 7).

## 4. Discussion

The use of a surgical method for treating a fracture of the mandibular condylar carries risk associated with the fixation material used. Complications that can occur after the use of osteosynthesis plates include screw loosening and plate fracture [51,52,53,54,55,56]. In order to maximize the chance of proper fracture healing in the postoperative period, numerous authors have been researching various methods of fracture fixation. The complexity of the problem can be seen in the multitude of types of plates available on the market (Table 1) for the fixation of mandibular condyle fractures.

The shapes of the plates used vary widely, from simple miniplates and miniplates with a reinforced bridge, used singly or two at a time, through square-shaped plates, to plates whose shape has been determined by the biomechanics of the mandible—trapezoid, delta, inverted Y, A-type, X-type or tau miniplates [57]. Studies on the rigidity of mandibular condylar process fracture fixation using different fixation materials have been performed by several methods. In the literature, there are studies of these plate shapes using finite element analysis, FEA [58,59], studies on animal mandibles [60,61,62], tests on polyurethane chaps [63,64] and clinical studies [65,66,67]. Due to the different natures of the aforementioned studies, their authors adopted different criteria for evaluating the plates tested. In the case of studies conducted on an animal model, the experiment consisted of loading an animal mandible with a certain force with fixation of a previously produced condylar process fracture and evaluating the displacement of the fragments. In the study conducted by Alkan et al. [60], the trial ended when the displacement reached 3.5 mm. In contrast, Pilling et al. [62] measured the force at which a loss of fixation stability would occur, which meant plate fracture, screw fracture, loss of screw stability or significant displacement. Studies using this method provide information on the force required for a given fixation to lose stability. It should be noted that the results obtained by this method in the form of force values may not match the conditions of osteosynthesis of condylar process fracture in humans due to the different anatomy of the mandible and the loading pattern of the condylar process area, which is different from the physiological one. Likewise, the results of our study do not inform about the clinical forces displacing the fragments, but only allow readers to differentiate between different plate designs. The authors of these studies report that polyurethane mandibles have similar strength parameters to human bone. In clinical studies, patients underwent radiographic evaluation after fracture osteosynthesis of the mandibular condylar process. In the study by Sugiura et al. [67], patients, six months after surgery, had a pantomographic examination and a posterior-anterior radiograph of the mandible to evaluate the proximal fragment medial flexion and vertical overlap of the fragments. The same radiographic studies were performed by Ahuja et al. In the study by Lechler [66], computed tomography was used for postoperative evaluation in addition to a pantomogram. In the case of studies conducted using the finite element method (FEA), the magnitude and direction of the displacement of the fragments relative to each other and the level of stress occurring in the plates and the bone adjacent to the plate, especially the bone surrounding the screws, are evaluated. Analysis of the results of tests performed using this method makes it possible to determine at what occlusal force the displacement of fragments relevant to the healing process or the loss of stability of the plate due to loosening of the screws can occur. Studies conducted by the finite element method (similar to the study on polyurethane models) provide constant test conditions for all tested variants of fixation of mandibular condyle fracture, so it is possible to reliably compare the tested plates in terms of osteosynthesis strength. The disadvantage is the considerably time-consuming nature of FEA, with the result that there are no comparative tests of more than a few plate types.

Each of the plates studied here can be used as a fixation material in the surgical treatment of fractures of the base of the condylar process of the mandible. Doubtless some plates would be better fitted for fractures running higher, and others for the fixation of fractures of the squat condylar process [3], but in general any can be used in this most common fracture of the condylar process of the mandible [2]. The comparison shows that the primacy of the most stable osteosynthesis has not changed. The best fixations mechanically are the double straight plates [68,69]. Two four-hole plates with a middle bridge are the state-of-the-art fixing material due to possibility of load sharing in an amount of 15.2 N in a one millimeter displacement. To reach this, round holes are needed for the screws in the 2.0 system. A detailed evaluation of the impact of compressible holes (i.e., oval-shaped) showed their adverse effect on fixation rigidity. The best are plates with seven, eight and nine hole/screws.

The following plates give the highest stability in the osteosynthesis of basal mandibular condyle fractures: TCP trapezoid pre-shaped 9, Endo-Condyle 25-283-25-91, XCP universal 3 + 5, XCP, XCP side-dedicated 3 + 5, TCP trapezoid TriLock 9 hole, XCP side-dedicated and ACP-T. The best mechanically are plates of large dimensions with a lot of round holes. The shapes of the holes significantly affect rigidity. For example, an XCP plate, if the design has only round holes, is one of the best mechanically. Adding two oval holes, on the lower part, nullifies this result and such a plate falls from the top to the middle of the peloton. A large number of holes worked well for TCP designs. Here, nine holes give excellent mechanical results. Transverse reinforcements of dedicated designs bring the mechanical strength closer to the two-plate ideal [28].

In verifying future plate designs for the fixation of mandibular condylar process fractures, the MEF is worth using. It is a combined measure of eight design features. Plate Design Factor used only four features of plate design and its evaluation loses the effect of oval holes on plate fixation rigidity. And it seems that the influence of oval holes has a negative influence on osteosynthesis rigidity [70]. The MEF values for a given plate design are strongly related to the measured average value of Fmax/dL (Table 1). Therefore, it seems to be a good tool to numerically describe the mechanical quality of plate designs.

Satisfied recovery, a key goal of precise fracture treatment, has become possible, as Artificial Intelligence (AI) technology in orthopedics has progressed in the following aspects: physical parameters can be predicted precisely through AI technology, bioactive implants made through three-dimensional printing technology can provide signals for endogenous repair and personalized surgical channels can be established through intelligent robotic technology. However, multilayer intelligent technology (MLIT) has not been used widely. With further study on biomechanical models for multiphysical parameter prediction, the stimulus response mechanism of bioactive implants and smart implants, the intelligent modeling of the 3D/4D printing process and variable structure in long-term therapy [71,72,73], satisfied repair may be achieved by MLIT [74]. This technology will be able to be assimilated into the best plate designs for the mandibular condyle in the future. By considering the advantages and disadvantages of plate design for the osteosynthesis of the mandibular condylar process base, Table 3 was constructed. An analysis of the best and worst features of osteosynthesis materials would likely provide valuable insights to improve the design, selection and implementation of the plates in condylar fractures. This information could potentially support improving patient outcomes, reducing complications and optimizing the overall effectiveness of the therapeutic approach.

Thanks to the skill of surgical teams, well-thought-out plate designs and reasonable surgical techniques in maxillofacial surgery, most fractures can be successfully treated at the primary and early secondary stages with plate and screw fixation [75]. This is possible even in major fragmented fractures, and most often it is not necessary to perform a total alloplastic joint replacement (as it is in orthopedics).

The present study is not a clinical answer to the question of effective osteosynthesis performed with specific types of plates, but rather a guideline for inventors for which direction to go in future projects. The reason for this is that, among other things, the fracture healing process depends on a great number of factors [26,76,77]. The composition of the materials used also affects the quality of the return and the condition of the patient [78,79,80,81]. These issues have not been addressed by the studies conducted, but future plate models with a high MEF can be selected for further development without conducting expensive strength tests. It is enough to analyze the series of plate design features that will be available from a known technical drawing. New plate designs are still appearing [57,82,83], and for this it will be worthwhile to compare them in a constant testing system.

## 5. Conclusions

The supremacy of the classic fixation of a mandibular condyle base fracture with two straight plates was confirmed. This is a type of osteosynthesis that will always find application and good results. However, it is noticeable that the large plate models give very similar osteosynthesis stabilities too, together with comfortable maneuverability.

Among the large number of described models of plates for the osteosynthesis of the mandibular condylar process, there is considerable variation in terms of the stability of the fixation performed with them. The proposed Mechanical Excellence Factor allows a pre-evaluation of the expected rigidity of fixation with a given plate without the need for a loading experiment. The authors expect this to be helpful in the future for inventors of new plates for the osteosynthesis of mandibular condylar process base fractures.

## Figures and Tables

**Table 1:** Compared plate designs placed from the weakest to the strongest fixing material.

Name	Design Code *	Design	H (mm)	W (mm)	S (mm^2^)	PDF	MEF	Fmax/dL (N/mm)
Grid Compression Plate	Plate 35	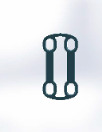	17.3	9.1	70	92	1.0	0.99 ± 0.39
Kolsuz’s Rectangular Plate	Plate 48	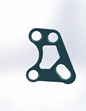	11.4	10.1	49	67	1.2	1.187 ± 0.21
Grid Plate	Plate 36	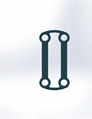	19	9.6	80	102	1.4	1.40 ± 0.38
Rectangle Plate 4 holes	Plate 32	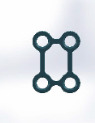	11	8.5	37	55	1.5	1.47 ± 0.16
Delta Condylar plate 3 oval holes	Plate 37	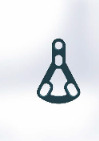	17	11.4	64	87	1.7	1.68 ± 0.63
Kolsuz’s Triangular Plate	Plate 47	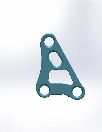	14	10.4	58	79	1.7	1.71 ± 0.12
Small Trapeze	Plate 33	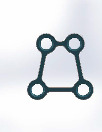	11.6	11.6	41	61	1.9	1.87 ± 0.21
Small Trapeze upper hole together	Plate 34	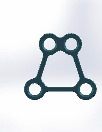	11.1	12.1	40	61	2.2	2.17 ± 0.32
Triangle lower 2 hole medially	Plate 45	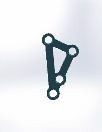	18.1	9.8	67	90	2.2	2.24 ± 0.38
Square Plate	Plate 31	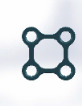	10	10	39	57	2.3	2.34 ± 0.66
Triangle lower 2 hole distally	Plate 42	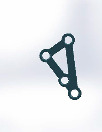	17.8	10	67	90	2.4	2.41 ± 0.54
Cut T-ACP	Plate 44	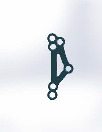	23.6	9.3	85	112	2.9	2.86 ± 0.37
Rectangle Plate 6 holes	Plate 38	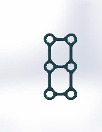	20	10	67	94	3.1	3.14 ± 0.63
Delta Condyle Compression Plate	plate 14	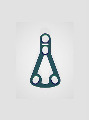	15.3	8.8	179	191	3.2	3.20 ± 1.39
Patent pending small triangle	plate 11	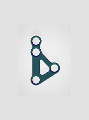	13.5	8	138	151	3.3	3.27 ± 0.36
Inverted Y	plate 28	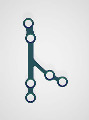	23.6	11.1	203	225	3.4	3.46 ± 0.51
Yang’s Keyhole Plate	Plate 46	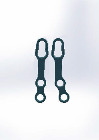	19.7	9,.9	80	106	3.,5	3.53 ± 0.81
Cut ACP-S	plate 08	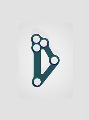	14.9	8.1	165	179	3.6	3.60 ± 1.29
XCP 3 + 5 with 2 compression holes	Plate 40	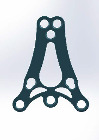	20.5	16	107	138	4.0	4.01 ± 0.88
Endo-Condyle 25-288-08-09	plate 21	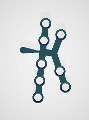	22.7	11	271	289	4.1	4.08 ± 0.46
Lambda	Plate 41	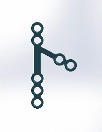	25.1	13.3	72	106	4.4	4.37 ± 0.41
Delta TriLock	plate 02	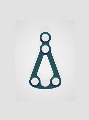	15.4	8.8	174	187	4.6	4.62 ± 0.90
TCP, trapezoid	plate 07	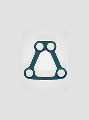	10.4	11.4	143	156	5.0	4.98 ± 1.42
Lambda Thinned	plate 01	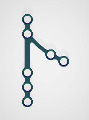	25.6	13	219	244	5.0	4.99 ± 2.46
A-Shape Plate	Plate 43	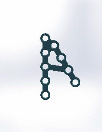	23.6	14.1	98	131	5.0	5.00 ± 0.62
Trapezoidal Plate	Plate 51	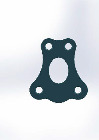	20	18	189	213	5.3	5.29 ± 0.67
Strut	plate 04	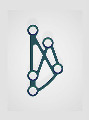	19	9.6	217	232	5.3	5.32 ± 0.47
Endoscopic Retractive 9 hole	plate 16	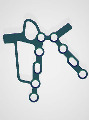	18	15.2	270	289	5.3	5.34 ± 0.80
Trapezoid Condylar Fracture Plate	plate 24	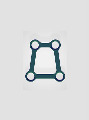	11.5	10.9	160	172	5.5	5.53 ± 1.35
TCP, anatomical TriLock	Plate 09	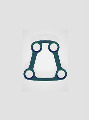	10.7	11.4	151	164	5.7	5.66 ± 1.13
PC7T	Plate 49	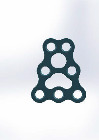	15	12,8	86	110	5.7	5.72 ± 1.41
Y geometry	Plate 50	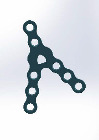	25	19	119	155	5.8	5.83 ± 0.52
PC5T	plate 06	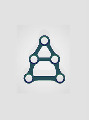	13.5	11.8	211	223	6.1	6.08 ± 1.00
Delta Plate	plate 27	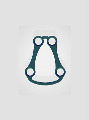	13.7	11.4	176	190	6.2	6.19 ± 1.02
Trapezoid Big	Plate 39	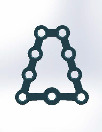	20.8	19.4	131	165	6.4	6.44 ± 0.45
Endoscopic Retractive 10 hole	plate 17	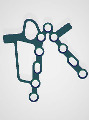	21.6	15.3	290	311	7.0	7.03 ± 0.56
PC7T modified	plate 03	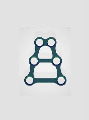	13.5	11.7	199	213	7.1	7.14 ± 0.89
ACP-VT	plate 12	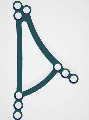	37	21	371	404	7.4	7.41 ± 1.97
Rhombic 3D—round holes	plate 26	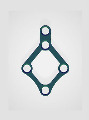	20	13	258	274	7.4	7.43 ± 0.67
Rhombic 3D—oval holes	plate 05	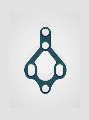	20	13	221	239	7.6	7.57 ± 1.60
cut ACP-T	plate 29	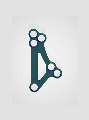	20	13	224	243	8.4	8.44 ± 2.25
ACP-S	plate 25	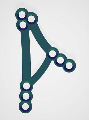	26	16.3	538	547	8.8	8.80 ± 1.14
TCP, trapezoid, pre-shaped 9	plate 15	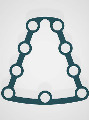	19.2	19.1	362	379	9.2	9.21 ± 1.77
Endo-Condyle 25-283-25-91	plate 30	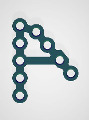	26.5	16.1	367	388	9.4	9.38 ± 2.13
XCP universal 3 + 5	plate 19	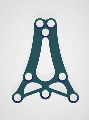	22.7	18	407	423	10.2	10.19 ± 1.65
XCP	plate 22	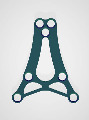	22.7	18	392	408	11.3	11.31 ± 1.41
XCP side-dedicated 3 + 5	plate 18	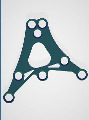	22.7	20	393	411	11.6	11.59 ± 4.03
TCP, trapezoid TriLock 9 hole	plate 13	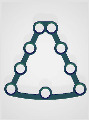	18.1	18.2	336	353	11.8	11.81 ± 2.28
XCP side-dedicated	plate 10	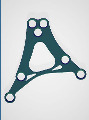	22.7	20	390	408	14.3	14.26 ± 0.99
ACP-T	plate 23	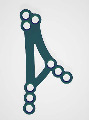	30.4	15	410	430	14.6	14.58 ± 2.60
Double Plain Plates	plate 20	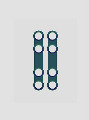	16.5	3.4	227	236	15.2	15.17 ± 2.69

* Names written with a lowercase letter correspond to the designs presented in an earlier publication [29], while names written with a capital letter are new designs. Abbreviations: H—height, W—width, S—surface area, PDF—Plate Design Factor, MEF—Mechanical Excellence Factor.

**Table 2:** Multiple range tests for force displacing the fixation by 1 mm (Fmax/dL, N) by plate.

Name	Mean Fmax/dL	Homogenous Groups ^1^
Grid Compression Plate	0.99	X
Kolsuz’s Rectangular Plate	1.19	XX
Grid Plate	1.40	XX
Rectangle Plate 4 holes	1.47	XXX
Delta Condylar plate 3 oval holes	1.68	XXX
Kolsuz’s Triangular Plate	1.71	XXX
Small Trapeze	1.87	XXXX
Small Trapeze upper hole together	2.17	XXXXX
Triangle lower 2 hole medially	2.24	XXXXXX
Square Plate	2.34	XXXXXX
Triangle lower 2 hole distally	2.41	XXXXX
Cut T-ACP	2.86	XXXXX
Rectangle Plate 6 holes	3.14	XXXXX
Delta Codyle Compression Plate	3.20	XXXXX
Patent pending small triangle	3.27	XXXXX
Inverted Y	3.46	XXXXX
Yang’s Keyhole Plate	3.53	XXXXX
cut ACP-S	3.60	XXXXX
XCP 3 + 5 with 2 compression holes	4.01	XXXXX
Endo-Condyle 25-288-08-09	4.08	XXXXX
Lambda	4.37	XXXXX
Delta TriLock	4.62	XXXXX
TCP, trapezoid	4.98	XXXXX
Lambda Thinned	4.99	XXXX
A-Shape Plate	5.00	XXXX
Trapezoidal Plate	5.29	XXXXX
Strut	5.32	XXXXX
Endoscopic Retractive 9 hole	5.34	XXXXX
Trapezoid Condylar Fracture Plate	5.53	XXXX
TCP, anatomical TriLock	5.66	XXXXX
PC7T	5.72	XXXXX
Y geometry	5.83	XXXXX
PC5T	6.08	XXXXX
Delta Plate	6.19	XXXXXX
Trapezoid Big	6.44	XXXXX
Endoscopic Retractive 10 hole	7.03	XXXX
PC7T modified	7.14	XXXX
ACP-VT	7.41	XXXX
Rhombic 3D—round holes	7.43	XXXX
Rhombic 3D—oval holes	7.57	XXX
Cut ACP-T	8.44	XXX
ACP-S	8.80	XXX
TCP, trapezoid, pre-shaped 9	9.21	XX
Endo-Condyle 25-283-25-91	9.38	XX
XCP universal 3 + 5	10.2	XX
XCP	11.3	XX
XCP side-dedicated 3 + 5	11.6	X
TCP, trapezoid TriLock 9 hole	11.8	X
XCP side-dedicated	14.3	X
ACP-T	14.6	X
Double Plain Plates	15.2	X

^1^ Multiple comparison procedure to determine which means are significantly different from which others. At the top of the page, 26 homogenous groups are identified from 51 plate designs using columns of Xs. Within each column, the levels containing Xs form a group of means within which there are no statistically significant differences. The method that discriminates among the means is Fisher’s least significant difference (LSD) procedure. With this method, there is a 5.0% risk of calling each pair of means significantly different when the actual difference equals 0.

**Table 3:** On the basis of the mechanical tests performed and the analysis of the Mechanical Excellence Factor, it is possible to indicate the unfavorable and favorable elements of the construction of the plate.

Structural Defects	Design Advantages
Small size	Big dimensions
Thin arms	Bulky plates
Few holes	More than 6 holes
Oval holes	Transverse arm connector
Long connectors between holes	Short connectors between holes

## Data Availability

The data on which this study is based will be made available upon request at https://www.researchgate.net/profile/Marcin-Kozakiewicz (accessed on 30 April 2023).
